# On the So-called Eczema Marginatum of Hebra (Tinea Trichophytina Cruris), as Observed in America—a Clinical Study

**Published:** 1877-11

**Authors:** L. Duncan Bulkley

**Affiliations:** Physician to the Skin Department, Demilt Dispensary, New York; Attending Physician for Skin and Venereal Diseases at the Ont Patient Department of the New York Hospital, etc.


### EXAMINER.

Vol. XXXV.—NOVEMBER, 1877.—No. 5.

### Original Communications.

### ON THE SO-CALLED ECZEMA MARGINATUM OF HEBRA (*TINEA TRICHOPHYTINA CRURIS*), AS OBSERVED IN AMERICA—A CLINICAL STUDY.

(Read at the First Annual Meeting of the American Dermatological Association, September 4th, 1877.)

By L. Duncan Bulkley, A. M., M. D.

Physician to the Skin Department, Demilt Dispensary, New York; Attending Physician for Skin and Venereal Diseases at the Out Patient Department of the New York Hospital, etc.

That there is a parasitic disease of the skin which shows individual characters, dependent on its peculiar location upon the thighs near the groin, there is no longer any doubt. The subject was thoroughly studied and discussed several years ago by Bärensprung, Hebra, Köbner, Pick and Anderson, and certain quite definite conclusions arrived at, which have been accepted by the majority of recent observers. It is my desire, in the present paper, to call attention to the disease as it occurs in this country, where it is not very infrequent, confirming the descriptions of others, as far as relates to the appearance of the eruption in New York city, where, however, it presents features very much less marked and severe, in many instances, than those described by writers in other countries; for I am not aware that it has ever been particularly touched upon by an American writer. I desire also to urge a plan of treatment which has been universally successful in my hands, but which is little known and hardly alluded to in the text-books.

This eruption was fully described in 1855 by Bärensprung[1], who found a parasite in all his cases, and who named the disease *herpes inguinum;* it must be mentioned that Devergie, in the preceding year[2] had mentioned it very briefly as a variety of herpes circinatus, or ringworm of the body. In 1860, five years after Bärensprung, Hebra, in the first edition of his book[3], gives exactly the same clinical picture under the name circumscribed eczema (das umschriebene eczem), or *eczema marginatum*, claiming that it is simply a variety of eczema; and this name, eczema marginatum, has since clung to the disease. Bärensprung was right in his perception of the true nature of the eruption and Hebra was wrong; the former died soon after and his description was forgotten, and the error in regard to it was not finally cleared up until nearly ten years later, when, after investigations by Pick[4], Hebra himself[5] ac-acknowledged the existence of a parasite, and afterwards, in the second edition of his book[6], he described it as *eczema marginatum parasitarium*, still treating of it as a variety of

1. Annalen des Charite Krankenhauses zu Berlin, VI Jahrg. Heft I., 1855, p. 150.

2. Devergie—Traité Pratique des Maladies de la peau, Paris, 1854, p. 274.

3. Handb. des Speciellen Pathol. und Therap. Virchow, Band. III, Hebra, 1860, p. 361.

4. Archiv. für Dermatologie und Syph., Vol. I., 1869, pp. 61 and 443.

5. Archiv. für Derm. und Syph., Vol. I, 1869, p. 163.

6. Lehrbuch der Hautkrankheiten, Zweite Auflage, Erlangen, 1874, Band. I, p. 485.

eczema. It may be added, however, that Köbner[1], in 1864, had found a parasite in eczema marginatum, had inoculated himself from it, and had, furthermore, claimed that the disease was but a herpes tonsurans of these parts; to this Hebra answered that the disease which he called eczema marginatum was a different affair, and that there was no fungus in it. This seemed to settle the question, until it was successfully re-opened by Pick in 1869, as before stated.

To show that the same affection was intended by each of these writers, as well as to furnish a means of comparison with the disease as observed here, I will briefly quote their descriptions:

Bärensprung says :[2] " There is an affection which has been generally known as hemorrhoidal erythema or eczema, which, although very common, has found hardly any mention in dermatological works. According to my experience, it is more common in men than women, and, as it appears commonly during the middle period of life, it is generally looked upon as a consequence of hemorrhoidal disturbances. It appears mostly in a limited area, embracing the external genital region and the anus, and is accompanied by a more or less severe itching. It occupies, by preference, that portion of the skin on the inner sides of the upper portion of both thighs which is in contact with the scrotum, and therefore almost always appears as if caused by pressure of the scrotum; it often extends on to the mons veneris, occupies the perineum and the region of the anus, and even the skin of the scrotum and penis itself.

" The diseased surface is sharply bounded by a curved or irregularly bent line, and the skin enclosed by it is red and rough, in many cases so dry that the disease resembles an erythema or a pityriasis; in other cases it appears excoriated, and to a certain degree moist. In still other cases the border is covered with small vesicles, or even small pustules, which

1. Klinische und exper. Mittheilungen aus der Derm. u. Syph., Erlangen, 1864, p. 6.

2. Annalen des Charite Krankenhauses zu Berlin, Bd. VI, 1855; Heft I, p. 150.

pour out a watery fluid which dries to thin crusts. In cases of this kind the accompanying itching is very severe, and the eruption appears more as an eczema or an eczema impetiginodes.

"The following are the grounds upon which," continues he, "I hold that this affection is essentially identical with the hitherto described forms of herpetic disease, and on which I class it as a herpes" (he meaning thereby our present *tinea circinata*):

"1. In all the cases I have found, in the epidermal scales and debris scraped from the affected portions, a vegetable parasite, that is, a branched and jointed mycelium which agrees perfectly with that found in the other forms. This vegetates in the superficial as well as in the deeper layers of the epidermis, in the root-sheath of the hairs, and attacks even the hairs themselves. I found the scrotal hairs brittle, and here and there filled with the neck-lace like threads of fungus, as seen in the hairs of the head in herpes tonsurans.

"2. The affection appears as a purely local one; it is not connected with any general disturbances, and is cured rapidly by the employment of purely local measures, and that without any injury to the general health.

"3. The disease has the same peripheral mode of extension as have the other herpetic affections.

"4. The disease appears in many cases to be communicated by contagion."

Without commenting on this for the present, other than to say that Bärensprung proceeds to detail a number of cases which furnish most perfect clinical corroboration of the above, I will at once quote Hebra's description, as it appeared in the first edition of his book in 1860[1], or fully five years after Bärensprung has published his article; Hebra's description is given without the slightest reference to the latter.

Hebra says, "By the designation eczema marginatum I indicate a peculiar form of eczema, which is separate and distinct from all other varieties of the disease, by its constant localization on the inner surfaces of the thighs, on the mons

1. Hebra, Acute Exantheme und Hautkrankheiten, 1860, p. 361.

veneris, and on the buttocks, by its peripheral extension and simultaneous clearing up of the center, by the distinct limitation of the periphery of the affected portions by an elevated line, on which the phenomena of eczema are especially developed, and finally by its almost exclusive occurrence in men, especially in shoemakers." "It always begins on the inner surface of the thigh, in men where the scrotum touches it, generally on the left side, there first appears a small, round, elevated spot which itches, is scratched and small punctate excoriations are seen. Shortly the center pales, so that only the red border is visible. Here we see sometimes papules, sometimes vesicles, sometimes excoriations, and later, in consequence of the drying of the exudation and the blood extravasated by scratching, small brown or black crusts are seen. The disappearance of the eczematous appearances in the center corresponds to the peripheral spreading of the disease, so that the originally small spot increases to the size of the hand. When it occupies this extent the central portions show considerable pigmentation, and the whole affected surface contrasts strongly with the healthy skin. Here and there in the central pigmented portion, single, small punctate, new developments of the eczema are seen, but the principal eczematous phenomena are observed on the outer edge." * * * * "The eczema seldom remains confined to the originally affected surface of the thigh; it generally spreads to the immediate neighborhood by the new development of the round patches, increasing to circles, or, the other thigh is affected symmetrically, the eruption gradually extending above, below, and behind, until, in untreated cases of long standing, the disease may reach to the navel, down to the knees and cover the buttocks." Hebra has also seen it extend to the back, breast, and neck, and once, on a female, even to the extremities.

Hebra at first questioned if this eruption were not an eczema modified by syphilis, but there is no mention in the first edition of his book of a suggestion occurring to his mind that it might be due to a parasite, which is a source of wonderment, considering how closely his clinical description corresponds with that of the behaviour of the vegetable parasitic eruptions, bearing in mind also Bärensprung's observations published five years previously.

There can then be no doubt that Bärensprung and Hebra were giving an account of the same affection, indeed the description of the latter seems almost as if taken from that of the former, so minutely do they describe the same appearances, in much the same words, a striking instance of the accuracy of the two brilliant clinicians, as Hebra does not seem to have seen the article of Bärensprung. In the second edition of his work he does refer to Bärensprung, not, however, to the description just quoted, but to a second article published in 1862, that is, *after* his own description had appeared; in this second article by Bärensprung the same disease is alluded to under the term "erythrasma," to which less than a dozen short lines are given. The object of this diversion is to give the credit to Bärensprung, to whom it belongs, as being the first to accurately and carefully describe the disease under consideration, he giving to it a name, herpes inguinum, which indicated its true nature (for by "herpes" Bärensprung and others of his time and later understood a vegetable parasitic disease, as it is now recognized to be); while the name eczema maginatum signifies nothing.

It is equally evident that Köbner in the work before referred to, intended the same affection, the clinical details of the first of his cases answering so perfectly to those given by Bärensprung and Hebra that it is needless to reproduce them here.

Finally, a number of Pick's cases were observed in the wards of Professor Hebra, and the diagnosis of eczema marginatum was made by him before Pick demonstrated the presence of the fungus parasite in them; their description, therefore, need not be given.

I cannot, however, forbear quoting here Devergie's brief description of the same eruption, written, as mentioned, in 1854, or the year before Bärensprung's article appeared, and six years before Hebra. Devergie, like Bärensprung, also classes it as a herpes; with herpes circinatus and herpes tonsurans, but he was not familar with the microscopic parasite, except by hearsay in regard to herpes tonsurans, and he does not suggest its existence in the affection under consideration.

Says he, [1] "There is one variety which is very commonly observed on the upper and inner surface of the thigh, in the neighborhood of the scrotum; it is a sort of a herpes circinatus in which the vesicles are more apparent and more secretory, and at the same time cause more burning and heat than is observed in the other varieties. The affection may readily be mistaken for intertrigo, for the contact of the scrotum with the thighs develops both affections. The existence of a circular, raised margin in this eruption, and its absence in intertrigo, distinguish the two affections apart, as does also the more abundant secretion in intertrigo. Herpes is a rebellious malady, in most cases easily reproduced."

To complete the bibliography of this discussion, mention should be made of the fact that McCall Anderson [2] in 1868 recognized and demonstrated the parasitic nature of this disease, and his description, as also his cases, answer in every way to the features of the disease as already detailed.

As illustrative of the clinical features of the eruption in this country, I will mention briefly twelve cases, of which I have full notes, all of which except two occurred in private practice, and in ten of which microscopic examinations were made, revealing the presence of the parasite in every instance.

Case I. A well known physician in New York aged, about 38, has worn a suspensory bandage for a varicocele for years; occasionally there has been some chafing of the thigh from it, but no serious skin trouble resulted until one month before his visit to me. He then noticed a small circular spot with well defined edges on the upper and inner surface of the left thigh, which continued to increase in size and to give more and more annoyance by its itching until he came under my care.

When first seen there were two rings, not circular, on the inner surface of the thigh, where the scrotum rested against

1. Devergie—Traité pratique des maladies de la Peau, Paris 1854, p. 274.

2. On the parasitic affections of the skin, London, 1868, 2d. edition p. 76.

it: one of them was about an inch in diameter, and the other one and a quarter by two inches. They were healed in the center, leaving a brownish and rather mottled skin, from which scales could be easily raised by moderate scraping. The margins of the eruption were very clearly defined, of a moderately active red color, composed in part of separated papules, but in the main the margin was unbroken; it was rapidly extending, leaving a cleared surface behind. This margin was distinctly, although very slightly, elevated. The side of the scrotum, (which lay against this surface in the night, unprotected by the suspensory) was red, itchy and presented some papules, which also could easily furnish scales on scraping.

Removing the scales and placing them in liquor potassæ and glycerine, on the slide of a microscope, magnifying two hundred and fifty diameters, they were found to present the mycelium and sporules of a fungus growth in great abundance, which resembled mainly the trichophyton tonsurans.

Sulphurous acid alone was prescribed, with directions to bathe and rub the parts freely with it, using it as strong as it was possible to procure it. The relief afforded was instantaneous and perfect, the language of the patient at the next visit being as follows: "You can hardly appreciate the exquisite relief given by the sulphurous acid when the itching comes on; it is perfect." Five days after the first application the margins of the eruption were still very well defined, much less elevated and with yet some scaling; the disease which had been rapidly spreading was completely arrested. Three weeks later, when last seen, the eruption on the thigh was well, the scrotum was still red, and covered with a glossy coating of cuticle, already separating, which had resulted from the too severe application of the sulphurous acid. From a letter still later, I learned that the eruption was cured at the end of one month from the first visit.

Case II. A. K. C., a large and somewhat fleshy gentleman, aged 30, first noticed a ring on the inside of the right thigh about three or four weeks before his first visit to me. On the following week a patch appeared on the opposite thigh, both of them being where the scrotum lies on the part. About the same time or shortly after, he found an eruption in both axillæ. He attributed the disease, with much probability, to a contagion acquired in Mexico, whence he had just returned, he saying that the filth of the country was such as to engender such diseases; he stated that it was a common custom there for the natives to wear the clothing of strangers sent to be washed, for a period before returning them; the clothes would often be retained thus two or three weeks before they could be gotten back.

When first seen the following conditions were recorded: On the right thigh was a very distinct ring, with a clearly defined margin, red, and slightly raised, of a line or two in breadth. The center of the patch, which was irregularly shaped and about four inches in diameter, was dry, of a dirty brown color, and slightly scaly, contrasting strikingly with the sharply cut margin; around the outside of the patch were a number of separate and isolated papules, flat and of a reddish color. On the left thigh there was a similar patch, but of much less extent. In the axillæ the eruption presented the usual appearances of ringworm of the body, and no other portions were attacked except those here mentioned; in the right axilla the disease occupied an area of about two inches in diameter; in the left axilla there was one small circle, about three-quarters of an inch in diameter.

The microscopic examination of the scales scraped from the margin showed a moderate amount of fairly luxuriant mycelium, and an abundance of spores. Sulphurous acid was ordered to be freely applied in full strength, and six weeks later he was next seen, after returning from a trip South, as purser of the Havana steamer. He reportel that the first application of the acid checked the eruption and arrested the itching at once, all of the cutaneous lesions disappearing entirely for a time. He then exhausted his supply of sulphurous acid, and had had none for twelve days, and the eruption had spread very luxuriantly during that time, especially during the last four days, which had been very warm and damp. There was now seen a large circle around the anus, occupying all the soft portion between the buttocks, with the same clearly defined, slightly prominent, red margin, and the dirty, yellowish-brown centre. It had also increased materially on the left thigh, and there was also the same on the left side of the penis and scrotum; under the arms the tinea was spreading, and on the right side of the neck there was a small, typical ringworm. The eruption about the anus was scraped, and the parasite found in the scales.

At a later date, two or three other spots of tinea circinata were seen; one on the left leg, near the ankle, and two on the left thigh, elsewhere than where the eczema marginatum existed in the crotch. As an adjuvant to the sulphurous acid, he was ordered the compound tincture of green soap, and an ointment of turpeth mineral, fifteen grains to the ounce, to be kept applied at night; it was to be washed off and the acid applied in the morning. Six days from the second visit the eruption had lost all of its distinctive characteristics; there was no more itching, and he went to sea again, apparently cured. He was directed to continue the use of the acid for a season, to prevent a relapse again.

Case III. J. E. S., aged thirty-five, was brought to me by his physician, during the intense heat of August, 1876, suffering terribly from what appeared to be an ordinary eczema of the whole region of the penis, scrotum, anus and abdomen. His history was, that he had had an eruption on these parts for at least three years, varying with the season and treatment, at one time better at another worse. He was also said to have had eczema of the ears five years or more ago, the remains of which had hung on ever since, in the way of scaling and some itching; he had also had what appeared to be eczema tarsi for a long time.

He was placed upon a treatment for his eczema of the genital region, consisting of alkaline and starch baths, dilute tar wash, followed by mild zinc ointment, and laxatives and alkalies. Very great relief was obtained, and, with certain other adjuvants, the case progressed very well, so that by the end of two weeks all, or most of the soreness was gone, the surfaces were dry, and the patient was quite comfortable. But still the disease seemed to remain in some localities, and resist various stimulant measures for eczema, although by constant employment of remedies, he was comparatively free from annoyance.

After all acute symptoms had long disappeared, two months and a half after his first visit, on examining the parts very carefully, or rather on taking a general look at the affected surface from a little distance, I noticed that there was a distinct red margin on both thighs, slightly raised, and of a color which contrasted strongly with the surrounding healthy tissues, and also with the skin enclosed by it, which was of a brownish-yellow. Placing some of the scales scraped off, beneath the microscope, they were found to be loaded with the mycelium and spores of a vegetable parasite; and certain hairs which were included in the specimen, were affected as in tinea tonsurans. This led to an investigation of the ear disease; and the scales taken from the auditory canal were examined, and found to contain fungus in abundance, the mycelium quite predominating, branching, often with enlargements and knobbed ends. In comparing the parasite with that seen in a fresh case of tinea tonsurans on the head of a child, which by good fortune appeared at the office on the same day, all the elements were found to be much smaller than those of the trichophyton, and corresponded more to the aspergillus. An examination of scaly crusts removed from the eyelids, showed also the presence of a parasite.

Here, then, we had the explanation of the chronicity both of the ear and eye trouble, and of that about the thighs. They had gotten better for a time under treatment for several years past, but the disease ever relapsed when not actively combatted, so that the patient, even when he considered himself comparatively well, had never been free from annoyance from the itching in the ears and about the thighs, and at times the distress had been very great. There had been the constant presence of the vegetable growths, which remained quiescent much of the time, but which always caused some irritation, and, when occasion invited, gave rise to much trouble. There can be little doubt but that the acute eczema of the penis, scrotum, and thighs, with which I saw him first, was the result of scratching; and when the irritation was, in a measure, relieved and scratching forbidden and prevented, the parts subsided to their chronic state in which the parasite was afterwards found.

Sulphurous acid was given him, when the parasitic nature of the affection was discovered, to be freely applied in full strength, and three days later it was recorded that there was very great change in the appearance of the thighs; all the marginated character of the eruption was gone. He had thought that there was comparatively little annoyance given by the disease at the previous visit, as it had been only what he had borne for several years, but on this occasion he expressed the greatest relief from that incessant irritation which had made him always conscious of the presence of the disease; the difference between his expressions before and after the use of the sulphurous acid was remarkable; he repeated so many times that he had experienced the greatest relief from it.

He was also ordered sulphurous acid, diluted with an equal part of glycerine, to be painted in the ear, and six days after its use it was noted that the ears were better than they had been for years. The eyes were bathed with a weak solution of soda, and a weak red precipitate ointment was used, to their great benefit.

There were some changes made in the treatment of the thighs, from time to time; occasionally the sulphurous acid would prove too severe, and an acute irritation would require other treatment for a while, but in the main the acid was persisted in, and at the end of about three months it was recorded that the thighs were entirely well; that the ears, which had given trouble incessantly for four or five years, were perfectly healthy, and that the eyes were in better condition than for many years. For the latter he wore glasses to correct an error in acccommodation, as suggested by Dr. Roosa, who saw the case with me and assisted in the treatment given to the eyelids and ears, concurring in regard to the parasitic nature of the eruption in these localities.

I may add that a number of times during the treatment of the case scales were scraped from the diseased surfaces, and the vegetable parasite was repeatedly found; also that in one axilla there was a quite distinct ringworm, of ordinary appearance. One more feature in reference to the eruption about the thighs was the occasional development of the same, sharply defined, flat, dull red papules of varying size, just outside of the ring of the so-called eczema marginatum, as was mentioned to occur in the first case, and occasionally the same within the area which had been passed over by the disease. These, as in the other cases, were considered to be new exhibitions of the parasite, and more assiduous application of the sulphurous acid caused their disappearance.

This patient remains under observation from time to time; I saw him socially a few weeks since, and continues free from the eruption which had annoyed him for years, the true nature of which had never before been suspected.

Case IV. Dr. Y., aged about 33, a physician of a neighboring city, consulted me in reference to an eruption which had existed a long while on the upper and internal surface of the right thigh. There was then a patch five or six inches long by one and a half to two inches wide, corresponding to the surface of contact of the scrotum and thigh. The margin of the surface was very sharply defined, being of a decided red color and slightly raised, with some outlying papules possessing the same features. The center of the patch was also somewhat reddened, the tissue slightly thickened and a moderate amount of scaling present, which could easily be increased by scraping. Microscopic examination showed abundance of spores filling the field, with some mycelial threads.

The history was that he had had irritation in this locality for several years, during which time he has had boils and carbuncular swellings on the scrotum; he has never been free from irritation and an eruption in this region, he says, for twelve years, although from his extreme cleanliness the disease has been kept within its present boundaries. The itching from it at times has been intense.

Sulphurous acid was advised to be freely applied, and by a letter I learned that it was followed with apparent benefit, but was discontinued after a few days in consequence of the distress to the respiratory passages caused by the fumes which arose from it. I have not seen him since, but he promised again to try the acid and report.

Case V. M. H. D., aged 29, presented the most marked example of a general eruption of tinea circinata which I have ever met with, and with it he had the marginated eruption about the genito-crural region. He had had the eruption for at least three years, and from the description of the case, recorded several years ago, it appears to have spread from below upwards, appearing first on the lower limbs. It had been located in the genital region for about two years previous to his visit; about a year after this it reached the neck, soon appeared on the face, and lastly on the left hand.

At his first visit the condition was noted as follows: The larger part of the body is covered with an eruption consisting of somewhat elevated, reddened circles, enclosing brownish-yellow, desquamating surfaces. Many of the circles are irregular, and in some instances quite broken, but the scaly surface exists almost everywhere, with occasional islands of unaffected tissue. The eruption really extends from the sole to the head, the face and neck having many ordinary ringworms upon them; the eruption had not extended into the hairy scalp except very slightly at the back of the neck, where the hairs were stumpy.

About the genital region the eruption possessed the characters described in the former cases of eczema marginatum—that is, on the sides of the thighs adjoining the scrotum were circles of reddened and slightly raised tissue, with moderate tendency to moisture, enclosing a brownish-yellow surface, slightly roughened. The itching and general irritation from the eruption was terrible; the patient walked around the office incessantly, being unable to sit long on account of the soreness of the buttocks from the disease.

His wife and children, he said, had all had attacks of ring worm.

Microscopic examination of the scales scraped from a number of places showed them to be loaded with the mycelium and spores of the trichophyton tonsurans, and the examination was repeated at a number of successive visits, always with the result of finding an abundance of the parasite. This was the first time the scales had ever been thus examined by a physician, and this the first suggestion of the true nature of the affection, although he had suffered from it for several years and had seen a number of physicians.

He was placed upon the pure sulphurous acid, to be freely applied, and in less than three weeks it was noted that the entire skin was nearly free from eruption. With the increase of warm weather in August the disease seemed to gain headway and again to give great annoyance; he was then ordered to use sulphurous vapor baths, several times weekly, and to continue the acid. By September 1st the baths had cleansed the skin greatly, but in spite of all treatment the parasite was still discovered in abundance by December 10th.

One year from the first visit he called to report that the eruption was about gone, there being, however, still a little on the neck, buttocks, feet, and left hand. He was in excellent spirits, the relief from the itching being very great; he was persisting in the use of the sulphurous acid and wished no other remedy to conquer the disease. That portion about the genitals ceased to give trouble very early in the treatment.

Case VI. M., aged about thirty, for four years had an itching eruption at the upper portion of the thighs and in the crotch ; it commenced after the repeated use of Turkish baths for several months, and had continued to spread ever since, the itching has been very great.

When examined the inner surface of both thighs for a distance of about six inches from the crotch, was the seat of a brownish, dry, scaly eruption, with a well defined, reddened edge ; the eruption extended back, well on to the buttocks. The condition of the scrotum was not noted. Examination with the microscope of scales scraped from it showed the abundant presence of fungus. He was placed upon sulphurous acid, but was seen only once and the result is not known.

Case VII. Mr. T. V. C., aged 54, first noticed a small red spot on the inside of the left thigh, where the scrotum touches it, four weeks previous to his visit to me. This continued to increase circumferentially, and other small circles appeared and developed, coalescing with the first one until his visit. It was recorded then that on the inside of the left thigh there was a patch of eruption about the size of the palm of a large hand, the center of which was healing, and the margin was distinctly defined and a little elevated. On the same side of the scrotum an exactly corresponding eruption was seen, the center of which was reddened and slightly scaly, and the margin was sharply cut, of a bright red, and moderately elevated. On the inside of the right thigh a single smaller spot had developed, and on that side of the scrotum the same was seen, in such a position that when the latter was allowed to rest on the thigh the two diseased spots exactly matched.

A microscopic examination of scales scraped mainly from the left side, revealed the mycelium and spores of a vegetable parasite, quite abundant.

He also was placed on sulphurous acid, which produced an immediate amelioration of the symptoms, but the skin proved very delicate and sensitive to the acid, and it was necessary repeatedly to suspend its use and adopt more soothing measures. There was also a considerable tendency to the development of boils in this case but the ultimate results were satisfactory.

CASE VIII. H. F., a large and rather fat German, fifty years old, appeared at Dr. Draper's Skin Clinic at the College of Physicians and Surgeons, on January 15th, 1877, for the relief of an eruption about the thighs and genitals. During my absence the following remedies were prescribed by one of the assistants, on the diagnosis of ordinary eczema, namely: two compound cathartic pills every second night, thirty grains of acetate of potassa, thrice daily, and the following ointment: ℞. olei cadini ʒij, pulv. camphoræ ʒiss, ung. simplicis ℥iss, glycerine ℥ss, M., to be well applied.

Two weeks after his first visit I saw him, and he reported that there had been no relief to the terrific itching which had so long distressed him. I then learned that the eruption was of several years duration, and had never yielded to any treatment, although he had been constantly under medical care.

His history is as follows : for ten or twelve years he has had chafing at the crotch during the hot weather, it going away with or without treatment on the approach of cooler weather (during about the same length of time he has become much more fleshy than previously). The present eruption did not begin, as the preceding, with warm weather, but, after the chafing of warm weather had ceased, in October, 1876, and increased instead of diminishing with the advent of cooler weather. At this date he first noticed an eruption on the upper part of the thighs, which gradually increased in severity under treatment until the date of his visit. The itching which he had endured he described as terrible, entirely depriving him of sleep; he would frequently get up in the middle of the night and walk several miles, and the parts bore testimony to his sufferings in the inflamed and irritated surface caused by his continual frictions.

When first seen the eruption resembled that in the cases already described, in many important particulars, but also presented points of difference. Both thighs at their upper and inner surface, to the crotch, were the seat of a red eruption, the outer portions more elevated than the inner, and the margins very well defined, and ended abruptly in healthy skin; there was no gradual shading into healthy tissue as in eczema. Around the outside of the principal portion were several smaller spots of disease of various sizes, from that of a pin's head to a third of an inch in diameter ; at late periods of the case there were more of these flat, out-lying papules, which I have recorded as existing in many of the other cases. The color of the affected surfaces was of a rather dusky red, the outer portions of a higher color than those toward the center ; the surfaces were moist, but this was largely from perspiration, as the surface could hardly be called a weeping one as in moist eczema. The sides of the scrotum were red and itchy. He also had chronic eczema on other parts of the body, behind the ears and on the hands.

The scales, scraped from the surface of the thighs, were examined and found to contain both mycelium and spores of a parasite abundantly, and the patient was placed upon the free external use of sulphurous acid. At the next visit, one week later, February 5th, I recorded that there had been a very manifest change in the appearance of the eruption, it had lost much of the elevated distinctness of its border, and, while it had been spreading rapidly before treatment, all advance had been arrested completely. But the statements of the patient afforded the most satisfactory proof of the correctness of the diagnosis. As before stated, he had not slept for more than two months previous to his visit; that night, after a thorough application of the sulphurous acid, he rested, sleeping all night, and had continued to experience the same relief up to the time of the note at the second visit.

To conclude the history of this case, he continued to improve week by week as my repeated notes show, until on March 5th, the marginated appearance was about gone, there was only a moderate redness of the parts, and no itching. On April 16th hardly any trace of the former difficulty remained. I have seen him on repeated occasions since, even during the present month of August, and he remains entirely free from his eruption; morever, this summer he has not even experienced the chafing which distressed him during previous warm seasons. From first to last he used only the sulphurous acid, no other remedy was necessary, and at no time was there any irritation of the skin requiring its cessation, as in several of the other cases.

Inasmuch as there was some little difference of opinion among some gentlemen who saw this case, I examined the scales from the surface several times, always with the result of finding more or less of the parasitic elements, until they had been removed by treatment. I also submitted some of the scales to my friend Dr. Heitzmann, who agreed with me in the existence of the parasite, and also as to the nature of the disease.

Case IX. Rev. Mr. B., aged about 40, has for several years suffered more or less from an eruption about the thighs and genitals, which has caused him to scratch much of the time, or rather has given an uneasy sensation which calls imperatively for scratching. On the inside of the right thigh was seen a reddish patch, three or four inches in diameter, bounded by an irregularly curved border, more reddened than the rest of the eruption, sharply lined toward the healthy tissue, the margin being decidedly more elevated than the central portion of the patch. On the left thigh, where the scrotum rested, was another smaller patch, possessing equally distinct characteristics. The surfaces were slightly scaly and on scraping them a fungus was found by the microscope to be present in a very easily appreciable amount. I find it recorded that the parasite was not seen when first looked for, until the scales had been soaked a little while in liquor potassæ and glycerine.

Case X. W. E., aged 33, said that two months previous to his visit to me an eruption developed on the inside of the left thigh, which "looked like ringworm," was circular, red, slightly elevated, and itchy. It had continued to increase in size, giving much disturbance, until he was sent to me for treatment. When first seen there was considerable artificial eruption mingled with the parasite disease, which former had been caused by a bi-chloride of mercury wash which had previously been advised by another physician. There was still, however, sufficient of the marginated appearance already described to warrant the diagnosis, and a microscopic examination of the scales demonstrated a parasite.

The case was treated for a while as one of ordinary eczema of these parts, because of the amount of irritation present, and at a later date the sulphurous acid was employed as in the other cases. One month after his first visit, a small circular ringworm developed on the inside of the right thigh, red and slightly elevated; this, he says, corresponded very closely with those which first appeared.

Case XI. Mr. C. S. D., aged 47, was seen recently, and but once, and the results of treatment are not yet known. About a year since he noticed an itching behind, about the anus, and three or four months ago the region of the scrotum and thigh began to give him trouble. He has also an eczema of the palms which has existed four years, and at times given him great annoyance.

Examination showed the following state of the parts. Around the arms a brownish, slightly roughened patch was seen, on both sides of the fold between the buttocks, limited externally by a distinctly drawn line of redder surface, which is slightly elevated and contrasts strongly with both the healthy skin and the enclosed brownish-yellow surface. On the left thigh, occupying a location corresponding to the place of contact of the scrotum, is a reddened surface of simple eczema, slightly moist, and without the marginated appearance; indeed, with the center of the patch more markedly affected than the periphery, a clinical sign, as we shall see, of some importance, pointing to an eczematous rather than a parasitic disease.

In this case also the parasite was found in the scales scraped from the region of the anus, upon careful microscopic examination, both spores and short mycelium. Sulphurous acid was ordered to the eruption about the anus, and other measures for the eczema existing elsewhere.

Case XII. A. B., a German woman, aged 36 years, was seen by me at the request of Dr. Hanks, in his room at the Demilt Dispensary, January 20th, 1877, and he kindly gave me the following notes of the case:—She had come to Dr. H. in August, 1876, for "an exceedingly troublesome and constant itching about the front passage, which had existed for six months or more. She was a stout, well-nourished woman, cleanly in her habits and person. On examination there was found an eruption covering the integument of the labia externa, perineum, and around the anus, showing frequent marks of the finger nails. The disease seemed to be very superficial, and the line of separation between healthy and unhealthy parts very marked. She was given benzoated oxide of zinc ointment with oil of cade, which gave no improvement, using also a vaginal wash of alum and chlorate of potash. An ointment of chloral and camphor, prescribed later, gave relief to the itching whenever applied, but at the end of several months the disease was about the same."

When I saw her, January 20th, the labia and to a slight extent the sides of the thigh, also the perineum, and the region of the anus were the seat of an eruption resembling the other cases of eczema marginatum here described. The area of disease was bounded by a very distinct margin, slighly raised, of a tolerably active red color, while within the surface was more of a yellowish-brown color, and not elevated as at the margin. The eruption extended around the anus, and as in the other cases the lines on the opposing soft parts of the buttocks corresponded exactly with each other in shape and contour.

I regret very much that the scales were not examined microscopically, but the clinical appearances were such that there was no doubt in my mind as to the parasitic nature of the eruption, and I therefore advised the free local use of sulphurous acid. Two weeks from that visit Dr. Hanks reports that the case was nearly well, and that she remained so, except around the anus, until a month or two ago, when she again complained; this time she had a crop of little boils, and more or less itching around the anus.

It is not my intention at the present time to attempt a complete study of this disease, with reference to the work of others; but I wish to bring forward these cases to illustrate the subject in some of its features as occurring in this country, for, unless familiar with it, the comparatively mild cases occurring here may escape attention, when they fail to present the severe features depicted by Hebra and described by others. I may add, that the cases here detailed do not constitute my entire experience in the disease, for each year I meet with a number of cases of it in public practice, of which I have no notes, and some of my cases in private practice may have been overlooked and 1 have the notes of a number of cases which I saw in the practice of the late Dr. H. D. Bulkley. The eruption is, undoubtedly, a somewhat rare one, but not nearly so rare, I believe, as some have thought it to be.

Diagnosis.—The only lesions which could be mistaken for *tinea trichophytina cruris*, the mis-called eczema marginatum, are eczema, intertrigo, and a serpiginous syphiloderm. The chief difficulty, of course, lies in the differentiation between simple eczema, or intertrigo, and the parasitic eruption, for the serpiginous papular eruption of syphilis needs only to be borne in mind, in this connection, to enable one to exclude it in any given case, with measurable ease. The microscope is, undoubtedly, of the highest value in the diagnosis; but ofttimes the disease has been so altered by treatment, that the parasitic elements can no longer be recognized, or the microscope may not be accessible, and, in any case, dependence must be placed largely upon clinical features.

In my experience, the itching in this eruption is far out of proportion to its apparent extent and severity; a patch which would be thought to give but little annoyance, will drive the patient almost frantic in his restrained desire to scratch; moreover, scratching and rubbing does not appear to give the relief afforded by the same in eczema, the tickling returns sooner, and is less completely removed by mechanical irritation. Eczema about these parts generally attacks the scrotum more severely than the thighs, and is more commonly attended with thickening of tissues in the former; whereas, in the cases of tinea of this region, when the penis or scrotum are involved, there is little or no thickening, except, perhaps, to a very moderate degree on the well defined margin; and the tendency of the parasitic disease is to attack the thighs far more than the penis or scrotum.

In eczema of the genito-crural region, as in eczema elsewhere, the tendency to recovery is from without inwards, toward the centre of the patch, whereas in the parasitic eruption the centre always tends to clear up first, although there may appear new developments within the healed area. Eczema shades off in many instances almost imperceptibly into the surrounding healthy skin, while in the disease under consideration, the sharply defined border, oftentimes quite perceptibly raised, has a characteristic appearance which, when once seen and clearly recognized, may be looked for and observed in all cases of tinea cruris which have not been too much altered by treatment. The dirty yellowish-brown color of the inner portions which have been passed over by the disease, are peculiar to the eruption as distinguished from eczema.

It is granted, of course, that in individual cases the element of eczema so far predominates that the correct diagnosis may be entirely impossible for a season, even to the practiced eye.

Thus, in Case III of our series, when the patient first came under observation, all the features of the eruption were those of an ordinary, very greatly aggravated case of common eczema of all this region; and the disease yielded, up to a certain point, to remedies directed against the eczema alone; and it was not until two months and a half after his first visit, when all the acute symptoms had subsided, and he considered himself about well, that the parasitic element was discovered. In this case, however, I believe that the tinea cruris was the original trouble which had pestered him for years, at times causing him to get up a severe eczema by scratching.

What is true in differential diagnosis of this eruption from eczema, is more especially true between it and erythema, and the features need not be repeated for that disease.

In regard to the use of the microscope in studying cases ot this disease, I do not think that the discovery of a vegetable parasite in the scales is always as easy a matter as it has been represented to be, nor should we, because we fail to find them in any one or two specimens scraped promiscuously from the affected surface, deny at once the parasitic nature of a case. That particular portion may be free from active vegetating elements, and yet contain seeds enough to cause it to break out again at a later period; also, we may not scrape deep enough, the outer layers in which it luxuriates most favorably may have been removed by treatment or by the cleanliness of the patient; or, again, the inflammatory phenomena may be so severe as to obscure the vision of the parasite, this is especially noticed by Kaposi.[1] This writer also gives, as far as I know, the only representation of the parasite growing in the deeper layers of the epidermis, even among the nucleated cells of the rete.

Neumann says,[2] "In an earlier stage of the disease, the fungus is almost always demonstrable ; in inveterate cases it is absent as a rule." "In those cases where the infiltration is considerable, and the skin covered with scales and crusts, I

1. Hebra—Lehrbuch der Hautkrankheiten. Vol. II., p. 641.

2. Neumann—Lehrbuch der Hautkrankheiten. 4th edit. Vienna, 1876. p. 639.

could rarely find anything else besides micrococcus and bacterium, with which the epidermal cells were densely packed. It appears that in this, as in all skin diseases dependent on fungus, and having thereby much dried blood and pus, the mycelium gradually perishes, and the cells of bacterium and micrococcus gradually preponderate, from which no higher forms are ever developed."

In the majority of cases, however, presenting clearly the clinical features detailed in this paper, the parasite can be found and demonstrated, if sufficient care and patience is exercised. It may, perhaps, be necessary to allow the disease to remain for a season without treatment to allow the free growth of the mycelium in the outer layers of the epidermis. There is always danger of confounding external elements of fungus with those belonging to the specimen under consideration, and great care is necessary to avoid this. It is also well to bear in mind that the edges of epithelial cells often resemble mycelium, and that fat globules have repeatedly been mistaken for the spores of a fungus.

Etiology.—This leads to a consideration of the etiology ot the disease. There is no doubt in my mind that it is frequently a combination of an eczema, pure and simple, or of an intertrigo, and a vegetable parasitic disease, in most instances the tinea trichophytina. I say in most instances, for I have seen cases of tinea versicolor, where the eruption extended as far down as the genital region, and where there were lesions resembling much the so-called eczema marginatum; and in Case III., before alluded to, the parasite appeared to be composed of finer elements than the trichophyton, and the co-incident occurrence of the parasitic disease of the ear, would point to the presence of the aspergillus in this case. I have once observed favus on the penis and scrotum, but it did not take the forms described in this paper, though I do not see why, if the person is a suitable subject, with an eczematous diathesis or tendency, this parasite might not luxuriate in the genital region, and give rise to the compound disease under consideration.

In a number of the cases here detailed, eczema existed elsewhere on the patient, but, on the other hand, in case V., where the very general tinea circinata covered almost the whole body, those portions about the genital region partook of the characters of eczema marginatum without there being any eczematous feature elsewhere; the favoring elements of heat and moisture in the parts affected appear to be the most important factors in the disease, besides the parasite.

It is, therefore, difficult to determine in every case how far the parasitic disease, and how far the eczema or intertrigo are to blame for the existing lesion, it is difficult to say which came on the stage first; I am inclined to believe that very frequently the parasite plays the second part in the programme, but soon becomes a prime factor—that is, that there is first an eczema or intertrigo, or a congested and moist condition of the parts upon which the fungus finds its most congenial nest where it vegetates and soon obtains a foot-hold, which nothing that does not exercise a directly destructive influence on its life can remove. Thus, in Case I., the patient had worn a suspensory bandage for a varicocele for several years, from which there had long been more or less chafing, but no serious trouble occurred until the advent of the parasite, a month before his visit. In Case VIII., the patient had had chafing in the crotch for ten or twelve years every summer; this had, however, always disappeared on the advent of cold weather; but when the vegetable parasite found lodgment here, on this appropriate soil, it remained until destroyed by a parasiticide. On the other hand, in a number of the cases, the eruption had persisted for years with aggravations, the disease being kept in check by cleanliness or treatment, or both, the parasite existing there all the time as a permanent cause, as in Case III.; in such cases the variation in the effect is dependent upon the state of the general health, congestion of the hemorrhoidal vessels, heat, exercise, etc.

It will be thus seen how inappropriate is the term *eczema* marginatum, while the addition of the adjective *parasitarium* but heightens the difficulty. It is no more appropriate to speak of a parasitic eczema than it is to speak of a syphilitic eczema; eczema cannot be caused by a parasite or by syphilis.

Prognosis.—I do not think that the prognosis of this disease, in this country, is anything like as serious as it has been spoken of by those who have written of it as observed elsewhere. Our cases are milder and certainly do yield to intelligent treatment in a manner which is generally highly satisfactory to the physician and patient. There is need of caution, however, lest the treatment be discontinued too soon, for relapses will happen unless the disease is thoroughly eradicated. Nor need we wonder at this, considering the extreme minuteness of the germinating spores of the fungus and the possibility, nay probability, of one or more of them being overlooked, perhaps remaining on articles of clothing, giving thus, in reality, a new infection, a new case. Caution should be exercised, therefore, in pronouncing the patient cured before he has remained some months free from eruption.

It must be borne in mind when comparing these cases with the severe ones alluded to by Hebra, McCall Anderson, Fox, and others, that they were all but two of them in private practice, and those two were of very cleanly habits; when comparing the eruption with the severe forms observed in the East, as the Chinese, Burmese ringworm, etc., we recall that the element of a warm climate certainly has an effect in the development of these affections (as several of my cases were decidedly worse in warm weather), and that diseases of the skin as well as other diseases, certainly do present differences of type in different countries, although some have tried to insist that they do not.[1]

Treatment.—The cases here given were treated almost exclusively by the free external use of sulphurous acid, as strong as could be procured and used, and, judging from the histories given, nothing more could be desired. In every case it gave immediate relief to the itching, and produced a marked improvement in the appearance, and in some instances it was the only remedy employed from beginning to end, and sufficed entirely to remove the disease.

Some care is necessary in order to procure and use sulphur-

1. Dr. G. Thin on Eczema Marginatum, London Practitioner, July, 1875, and Jan. 1877.

ous acid strong enough. I have often seen recommendations of writers to employ it diluted in various strengths, but seldom have I found it necessary to weaken it any, even for delicate skins, while I constantly see it fail in its action from being too weak. I generally advise patients to buy an unopened bottle of Squibb's sulphurous acid, to open it carefully and fill a one ounce bottle, cork again quickly, and make all the applications from the smaller bottle, which is to be refilled as often as required. I am convinced that much of the sulphurous acid retailed in the shops is absolutely worthless, as the gas either has evaporated, leaving simply the distilled water, or the sulphurous acid held in solution in the water has partially undergone oxidation, returning to sulphuric acid in solution, which, in place of being beneficial, would be prejudicial to such an eruption as the one under consideration. It is necessary, then, to use freshly made acid which has been kept tightly corked, and as this has been my habit for several years, I seldom find difficulty in obtaining the results I desire from it in vegetable parasitic affections of the skin.

In regard to the mode and frequency of its use, I advise the parts to be well saturated with it two or three times a day, there is no harm in applying it much more frequently if it does not inflame the skin, indeed many patients resort to the application of it whenever the itching calls attention to the disease. When it appears to cause irritation it is to be suspended, and such remedies as zinc ointment, or a calamine lotion are to be employed for a time, and on returning to the sulphurous acid, a new and, if practicable, an unopened package, fresh from the manufacturer, should be employed, as possibly the irritation may have been caused by the *sulphuric* acid, developed in the former specimen.

I need hardly mention other methods of treatment which are familar to all. Anderson says he has had best results from the bi-chloride of mercury in solution (gr. ij. ad. ℥j.) ; others recommend ointments of turpeth mineral (gr. xv. ad. ℥j.), or dilute citrine ointment, as also oil of cade, oleum rusci, etc. Goa powder, or poh di bahia, or its derivative crysophanic acid, is a paraciticide of value, especially in this form of disease, and is highly recommended by some. Hebra and Neumann treat this disease much as they would ordinary eczema of these parts, although recognizing its parasitic nature ; this I believe to be a great error, for while vigorous treatment may succeed in removing the disease mechanically, the results are far less rapid and satisfactory, in my experience, than those of a well directed anti-parasitic plan, and, on the other hand, I may say that most of my patients had been under judicious treatment for eczema with little or no avail, until the real nature of the affection was discovered, and it treated in the method described.

Hebra[1] recommends his modified Wilkinson's ointment (℞. sulph. flor. picis liquidæ, aa ℥iij. saponis viridis, unguent. simpl. aa ℥vi. cretæ prep. ʒij. M.) to be rubbed in with a brush morning and night for six days, and the parts kept covered with flannel. The surface is not to be washed during this time, nor for three days afterwards ; a bath is then taken. This treatment generally confines the patient to the house, or even to the bed ; when this cannot be done Hebra states that any other treatment must be mainly palliative, and must last for months. In the use of the sulphurous acid I have never seen the patient confined to bed, the treatment is measured generally by days or at least weeks, and not by months.

I have sometimes obtained considerable assistance from an occasional sulphur vapor bath, which, of course, is but a part of the sulphurous acid treatment, in the one instance the sulphur fumes being dissolved in water, in the other they are directly applied to the skin. I have not used sulphur otherwise, as in ointment, nor should I be disposed to try it from what I know of the disease.

---

(1.) Hebra—Lehrbuch der Hautkrankheiten—Erlangen 1874—Zweite Aufl. p. 493.

